# Comparison among three variant callers and assessment of the accuracy of imputation from SNP array data to whole-genome sequence level in chicken

**DOI:** 10.1186/s12864-015-2059-2

**Published:** 2015-10-21

**Authors:** Guiyan Ni, Tim M. Strom, Hubert Pausch, Christian Reimer, Rudolf Preisinger, Henner Simianer, Malena Erbe

**Affiliations:** Animal Breeding and Genetics Group, Georg-August-Universität, Göttingen, Germany; Institute of Human Genetics, Helmholtz Zentrum München, Neuherberg, Germany; Chair of Animal Breeding, Technische Universität München, Freising, Germany; Lohmann Tierzucht GmbH, Cuxhaven, Germany; Institute for Animal Breeding, Bavarian State Research Centre for Agriculture, Grub, Germany

**Keywords:** Whole-genome sequencing data, Variant calling, Imputation accuracy, Layer chicken

## Abstract

**Background:**

The technical progress in the last decade has made it possible to sequence millions of DNA reads in a relatively short time frame. Several variant callers based on different algorithms have emerged and have made it possible to extract single nucleotide polymorphisms (SNPs) out of the whole-genome sequence. Often, only a few individuals of a population are sequenced completely and imputation is used to obtain genotypes for all sequence-based SNP loci for other individuals, which have been genotyped for a subset of SNPs using a genotyping array.

**Methods:**

First, we compared the sets of variants detected with different variant callers, namely GATK, freebayes and SAMtools, and checked the quality of genotypes of the called variants in a set of 50 fully sequenced white and brown layers. Second, we assessed the imputation accuracy (measured as the correlation between imputed and true genotype per SNP and per individual, and genotype conflict between father-progeny pairs) when imputing from high density SNP array data to whole-genome sequence using data from around 1000 individuals from six different generations. Three different imputation programs (Minimac, FImpute and IMPUTE2) were checked in different validation scenarios.

**Results:**

There were 1,741,573 SNPs detected by all three callers on the studied chromosomes 3, 6, and 28, which was 71.6 % (81.6 %, 88.0 %) of SNPs detected by GATK (SAMtools, freebayes) in total. Genotype concordance (GC) defined as the proportion of individuals whose array-derived genotypes are the same as the sequence-derived genotypes over all non-missing SNPs on the array were 0.98 (GATK), 0.97 (freebayes) and 0.98 (SAMtools). Furthermore, the percentage of variants that had high values (>0.9) for another three measures (non-reference sensitivity, non-reference genotype concordance and precision) were 90 (88, 75) for GATK (SAMtools, freebayes). With all imputation programs, correlation between original and imputed genotypes was >0.95 on average with randomly masked 1000 SNPs from the SNP array and >0.85 for a leave-one-out cross-validation within sequenced individuals.

**Conclusions:**

Performance of all variant callers studied was very good in general, particularly for GATK and SAMtools. FImpute performed slightly worse than Minimac and IMPUTE2 in terms of genotype correlation, especially for SNPs with low minor allele frequency, while it had lowest numbers in Mendelian conflicts in available father-progeny pairs. Correlations of real and imputed genotypes remained constantly high even if individuals to be imputed were several generations away from the sequenced individuals.

**Electronic supplementary material:**

The online version of this article (doi:10.1186/s12864-015-2059-2) contains supplementary material, which is available to authorized users.

## Background

The technical progress in the last decade has made it possible to sequence millions of DNA reads in a relatively short time frame for reasonable costs. Thus, whole-genome sequencing has become available that allows us to gather more information on genetic variation, genes, gene function and other characterizations of genomes [1, 2] and the number of research projects dealing with whole-genome sequencing data has been emerging in humans [3–6], domestic animals [7–9] and other species [10] in the last years. Large consortia (e.g. 1000 bull genomes project [9, 11] or the human genome project [12, 13]) have been established to accumulate available resources, detect new variants in genomes, better understand genetic architecture of different traits and find or narrow down positions of potential causal loci. In dairy cattle, for example, 28.3 million variants in the whole genome were identified from 234 bulls sequenced with an average coverage of 8.3X in the first phase of the 1000 bull genomes project, and loci associated with milk production and curly coat were detected by genome-wide association studies [9]. In chicken, research projects using whole-genome sequencing data have been rare so far. Rubin et al. [8] generated pooled whole-genome sequencing data representing eight populations of domestic chickens in order to identify how genetics adapt to new environments. In the study of Qanbari et al. [14] genome regions with strong evidence of selection were identified from pooled whole-genome sequencing data of 15 laying chickens. Within the framework of the project Synbreed (http://www.synbreed.tum.de/) whole-genome sequencing data of 50 individuals from commercial layer lines were generated which built the basis for this study.

Several variant callers based on different algorithms have emerged using single or multiple samples simultaneously, e.g. SAMtools [15] or GATK [16]. Recently, some studies have shown that there is significant difference in the set of variants called by different variant callers [7, 17, 18]. Baes et al. [7] found that the number of variants varied between variant callers (i.e. Platypus, SAMtools and two difference GATK utilities: UnifiedGenotyper and HaplotypeCaller) in whole-genome sequencing data of dairy cattle. O’Rawe et al. [18] carried out a study to examine the concordance among different variant calling pipelines with default parameters, but their analyses mainly focused on exome sequencing and did not assess multiple sample variant calling algorithms. Thus, it is still important to evaluate genotype concordance and precision obtained with different variant callers in whole-genome sequencing data in chicken.

Although over the past several years the cost of DNA sequencing has decreased by several orders of magnitude due to the rapid development of sequencing technology, it is still comparatively expensive [19]. There are two main strategies to reduce costs: One is to only sequence coding exons which has been commonly used in human clinical applications [20], but actually none of the available kits can cover all the coding exons [21]. Besides, it was shown that both natural and positive selection eventually occurred in the non-coding DNA blocks and some QTL have been mapped in such blocks, so that important parts of the genome may be missed by just using exome sequencing [22, 23]. The other major strategy to reduce costs when being interested in sequence information of a whole population is to generate whole-genome sequencing data for a small set of individuals highly related to the population and then impute SNP array data of other individuals of the same population up to sequence level based on the whole-genome sequencing data of the sequenced individuals and array based SNP array data of the remaining individuals. Before whole-genome sequencing data had been available, the technique of imputation has already been used for imputing from low to high density SNP array data with high accuracy and thus has proven to be a successful line of action (e.g. in cattle [24]) to obtain higher marker densities for a large number of individuals.

Heidaritabar et al. [25] showed the possibility to impute SNP array data into whole-genome sequencing data based on a small reference population of 22 sequenced individuals in simulated data. Druet et al. [26] investigated the accuracy of imputation that can be achieved with Beagle [27] and found that the highest imputation accuracy was 0.86 when the simulated whole-genome sequencing data for 50 bulls with a 12X coverage was used as reference dataset. Van Binsbergen et al. [28] and Pausch et al. [29] showed that a reasonable accuracy of imputation (e.g. correlation between observed and imputed genotypes as high as 0.83) could be achieved when imputing from SNP array data to whole-genome sequencing data in dairy cattle breeds. Nevertheless, there has been no attempt so far to evaluate the accuracy of imputation from high density SNP array data (580 k) up to sequence level with real chicken data.

In this study, we first compared the sets of variants detected with different variant callers, namely GATK [16], freebayes [30] and SAMtools [15], and checked the quality of genotypes of the called variants in a set of 25 white layer and 25 brown layer individuals. Second, we assessed the imputation accuracy from SNP array data to whole-genome sequencing with three different imputation programs, namely Minimac [31], FImpute [32] and IMPUTE2 [33], in a brown layer line.

## Methods

### Ethics statement

Samples were collected by veterinarians in the Lohmann Company in the course of a routine health check for diagnostic reasons and a partition of retained samples was used to extract DNA. The authors collected no samples themselves.

### Data

Blood samples and pedigree data of more than 5 generations backwards (2260 individuals in total) were available for purebred individuals from different generations of a brown layer line. Number of individuals per generation is shown in Additional file 1. Furthermore, genotypes from the Affymetrix Axiom® Chicken Genotyping Array (580 k array) were available for 1081 brown layer chickens (including 24 of the 25 sequenced brown layers) from 5 different generations which were later imputed to whole sequence level. Genotyped SNPs with minor allele frequency (MAF) smaller than 0.5 % and genotyping call rate smaller than 97 % were removed so that 350,602 SNPs remained. Individuals with a call rate smaller than 95 % in the remaining SNPs were then excluded leaving a set of 1075 genotyped brown layer individuals.

### Whole-genome sequencing and alignment

Fifty individuals (25 brown layers and 25 white layers) chosen to be from one of the older generations and highly related to the set of already genotyped individuals were sequenced with the Illumina HiSeq2000 technology with a target coverage of 8X. Sequence reads were aligned to Build 4 of the chicken reference genome (galGal4) using BWA (version 0.7.9a-r786) [34] with default parameters for paired-end alignment. In this step SAM files were generated, which were converted to BAM files using SAMtools [15] in the following step. Reads were then further processed with the MarkDuplicates utility of Picard (http://broadinstitute.github.io/picard/) to remove potential PCR duplicates.

### Variant detection

Variants including SNPs and short insertion and deletion (INDELs) were called using different software programs: GATK (version 3.1-1-g07a4bf8, UnifiedGenotyper) [16], freebayes (version v0.9.15-1-g076a2a2) [30] and the mpileup utility of SAMtools (version 0.1.19-96b5f2294a) [15] with default parameters, respectively. With all programs mentioned, variant calling was performed with multi-sample approaches using all 50 sequenced chickens simultaneously. Sets of variants obtained with the three different callers were processed in equal manner, but independent from each other in the following. Different versions of the same variant callers may result in a different set of variants for the same underlying sequencing data. Thus, two versions of freebayes (version v0.9.15-1-g076a2a2 and version v9.9.2-22-gc283d6d) were compared regarding the overlap of called variants.

### Filtering and genotype quality enhancement

To reduce the proportion of the false positive variants, different strategies to select the so-called high-quality variants have been suggested [14, 18, 35, 36]. We applied thresholds for depth of coverage (DP) and mapping quality (MQ) according to the following protocol: Extraction of SNPs from the set of all called variants was done using the SelectVariants command of GATK. Filtering for the SNPs called on all chromosomes of the whole-genome included the following criteria: First, outlier SNPs (top 0.5 % of DP) were removed. Then, mean and standard deviation of DP of the remaining SNPs were calculated and SNPs with a DP above and below 3 times the standard deviation from the mean were removed as well. For mapping quality, SNPs with a MQ score smaller than 30 within SNP sets obtained with the variant callers GATK and SAMtools and SNPs with both mean mapping quality of observed alternate alleles (MQM) and mean mapping quality of observed reference alleles (MQMR) smaller than 30 within the SNP set obtained with freebayes were excluded from further analyses. Separate SNP sets were built for brown and white layers, respectively, in which SNPs that were monomorphic in the respective set of individuals were removed. Finally, we used Beagle 3.3.2 [27] (see Additional file 2 for the pipeline) in order to enhance the original genotype quality of the remaining SNPs following the proposal of Jansen et al. [37]. For all subsequent analyses regarding imputation, only data from brown layers and variants called by GATK were used. Furthermore, considering the computational efforts especially in the imputation process, all further analyses were not performed for the entire genome, but three chromosomes (chromosomes 3, 6 and 28) of different length were selected for the following analyses.

### Validation of different variant callers

Genotype concordance (GC), non-reference sensitivity (NRS), non-reference genotype concordance (NRC), and precision were calculated based on array genotypes and corresponding sequence-based genotypes obtained with different variant callers (GATK [16], freebayes [30], SAMtools [15]). For each SNP, GC is the proportion of individuals whose array-derived genotypes are the same as the sequence-derived genotypes over all non-missing SNPs on the array. NRS is the number of individuals who have at least one non-reference allele in both whole-genome sequencing data and SNP array data divided by total number of individuals who have at least one non-reference allele in the array data. NRC is the number of animals whose array-derived genotypes are the same as the sequence-derived genotypes and are not homozygous for the reference allele divided by total number of individuals who have at least one non-reference allele in the SNP array data. Precision is the number of animals whose array-derived genotypes are the same as the sequence-derived genotypes and are not homozygous for the reference allele divided by total number of individuals who have at least one non-reference allele in the sequencing data. The detailed calculations are shown in Additional file 3, which were based on the definitions in DePristo et al. [38] and Linderman et al. [20]. Validation of variant callers was done for all positions at which SNPs from the array were available on chromosomes 3, 6 and 28 (34,311, 13,627 and 2,730 SNPs), respectively, for the 24 brown layer individuals that were both genotyped and sequenced.

### Imputation

Imputation was done with three software packages: Minimac [31], FImpute [32] and IMPUTE2 [33], among which Minimac and IMPUTE2 are based on pedigree-free algorithms, while FImpute can combine linkage disequilibrium (LD) information and pedigree information in the imputation process. FImpute uses an overlapping sliding window method to detect the relationship between study and reference set, while IMPUTE2 apples a hidden Markov model. Minimac implements the MaCH [39] algorithm for genotype imputation. For all software, a default number of iteration was used. As Minimac and IMPUTE2 need phased input data, pre-phasing for whole-genome sequencing and SNP array data was performed using Beagle 4 [27].

### Assessment of imputation quality

To evaluate the accuracy of imputation from SNP array data to whole-genome sequencing data, three strategies (described below) were used.

#### Leave-one-out cross-validation

Since 24 out of the 25 sequenced brown layer chickens were also genotyped with the 580 k array, each of these individuals was excluded from the imputation reference data set once and imputation from SNP array data to whole-genome sequencing data was performed with the respective individual being one of the validation individuals in the resulting dataset. Genotype concordance and correlation between the imputed and sequenced genotypes from these run for all non-monomorphic SNPs being not on the array was calculated afterwards per the respective individual.

#### Father-progeny pair conflicts

Among the genotyped brown layer individuals there were 134 individuals that were progeny of one of the sequenced individuals. Thus, genotypes on imputed SNPs in the progeny could be compared to the father’s genotypic information at these SNP positions and genotype conflicts (alternative homozygotes in father and progeny) were counted. Proportion of genotype conflicts were calculated per father-progeny-pair over all SNPs excluding the ones which were also genotyped using the 580 k array on chromosomes 3, 6 and 28, respectively.

#### Accuracy of randomly masked 1000 SNPs

As imputation accuracy depends (amongst others) on the degree of relationship between sequenced individuals and individuals to be imputed, we also checked how imputing accuracy changes when different numbers of generations are between sequenced individuals and individuals to be imputed. For this analysis, we randomly masked (i.e. setting them to missing) 1000 SNPs (680 out of total 34’311 SNPs on chromosome 3, 270 out of 13’627 on chromosome 6 and 50 out of 2736 on chromosome 28) from the SNP array data in all genotyped individuals and imputed those SNPs as if they were SNPs from the sequence data. Afterwards, imputed genotypes on these 1000 SNPs and real array genotypes were compared and genotype correlation was calculated for each SNP and also for each individual. As Calus et al. [40] and Mulder et al. [41] demonstrated that it is better to center and scale true and impute genotype when calculating the individual-specific imputation accuracy, we investigated the individual-specific imputation accuracy based on original genotypes and standardized genotypes. The random masking was replicated five times with different random sets of 1000 SNPs and means of these five replicates are reported in the results.

## Results and discussion

### Alignment and coverage

For brown layer chickens, on average 88 million paired-end reads were obtained per individual. Among these reads, 1.72 % (ranging from 0.76–1.98 %) on average were marked as duplications and excluded and on average 96.7 % (ranging from 96.1–96.9 %) were mapped against the reference genome (galGal4). Coverage per sample ranged from 5.0 to 16.6, with an average of 7.6. For white layer chickens, on average 94 million paired-end reads were obtained per individual. Among these reads, 1.69 % (range 1.45–1.92 %) were marked as duplications and excluded; and 96.7 % (range 96.3–96.9 %) were mapped against reference genome. Coverage per sample ranged from 7.9 to 15.6, with an average of 10.8. Based on this data set, the number of raw paired-end reads obtained was higher in white layer chickens than in brown layer chickens. However there was no difference in percentage of duplications and percentage of mapping. Details can be seen in Additional file 4.

### Variant detection

Depending on the variant caller, totally 13,442,923 (freebayes [30]), 13,642,483 (SAMtools [15]) and 14,757,670 (GATK [16]) variants (i.e. SNPs and INDELs) were detected with multi-sample calling on the 50 available brown and white layer chicken genomes (Additional file 5). In the study of Cheng et al. [36] GATK identified almost the same amount of variants as SAMtools, while Pattnaik et al. [42] identified more SNPs with freebayes than with SAMtools or GATK in the human genome. Unlike their results, we found that GATK identified more variants than SAMtools and freebayes, thus showing the same tendency as in studies of Liu et. al [43], O’Rawe et al. [18] and Baes et al. [7]. On the three chromosomes 3, 6, and 28 selected for imputation, GATK identified 2,297,603 variants per animal of which 2,054,930 were SNPs. After excluding low-quality SNPs that did not match the filtering criteria (as defined in the method section), there were 2,021,911 SNPs remaining. Compared to GATK, both SAMtools (2,125,837) and freebayes (2,055,976) detected less variants, and after excluding INDELs and filtering, there were 1,759,887 (1,652,870) SNPs remaining with SAMtools (freebayes).

**Fig. 1:**
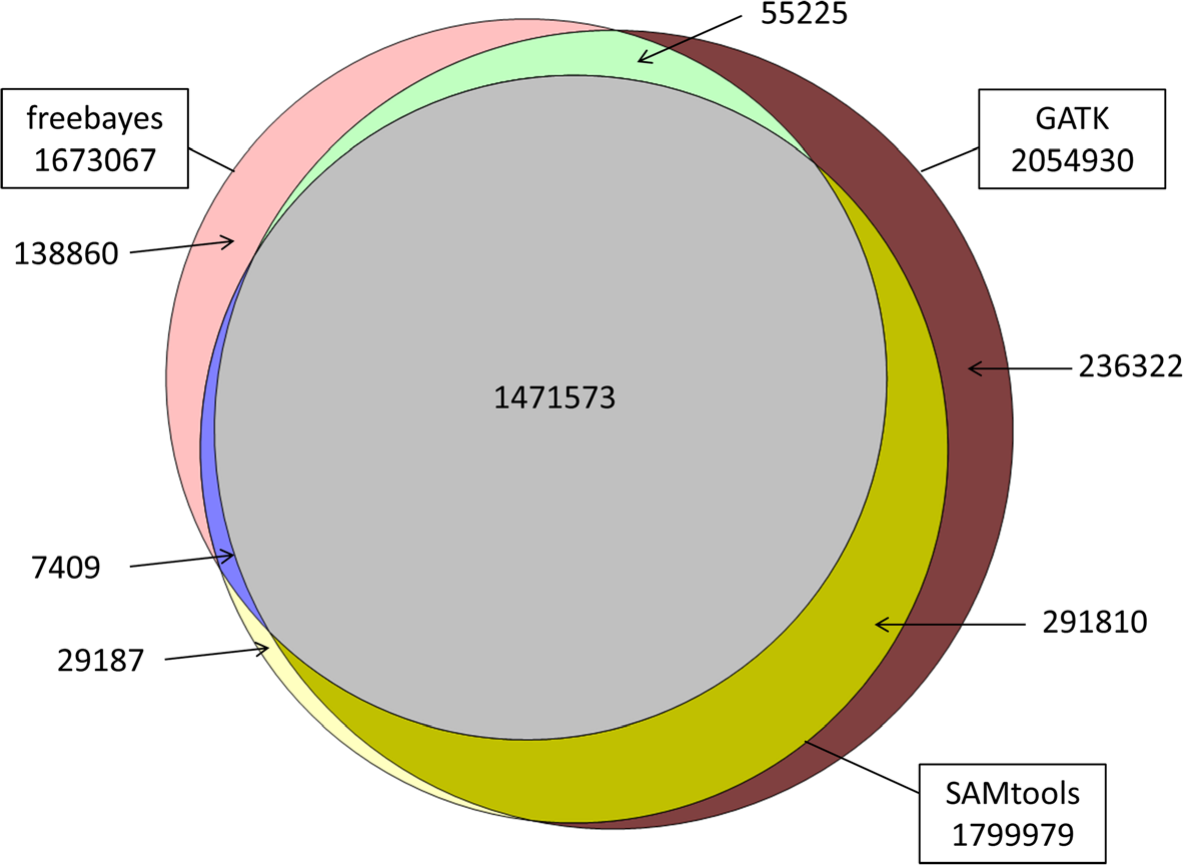
The overlap of single nucleotide polymorphisms detected by different variant callers

Figure 1 illustrates the number of overlapping SNPs detected by the three variant callers on the three chromosomes 3, 6 and 28. Many SNPs were detected only by one variant caller (236,322 for GATK, 29,187 for SAMtools, and 138,860 for freebayes). However, 1,471,573 SNPs were detected by all three callers, which is 71.6 % (81.6 %, 88.0 %) of SNPs detected by GATK (SAMtools, freebayes) in total. When focusing on GATK and SAMtools only, 1,763,383 SNPs were detected by both of them, which were the 86 % of total SNPs detected by GATK, and 98 % of total SNPs detected by SAMtools. Baes et al. [7] found that around 18.3 million SNPs were both detected by SAMtools and GATK in the whole-genome of 65 individuals of the Swiss dairy cattle population, which was 83 % of the total number of SNPs detected by GATK and 98 % of SAMtools which are very similar proportions to the ones we observed in our study. Based on data from exomes of 20 humans, Liu et al. [43] found in an exome sequencing study that 23,824 SNPs were both detected by SAMtools and GATK, which is 95.5 % of the total number of SNPs detected by GATK and 89.8 % of SAMtools. A high agreement of different callers (i.e. a high percentage of SNPs detected by different callers simultaneously) with each other in terms of called variants, is an advantage if whole-genome sequencing data is handled in a way like O’Rawe [18] suggested, namely using only the variants discovered by multiple variants callers or pipelines for further analyses.

**Fig. 2:**
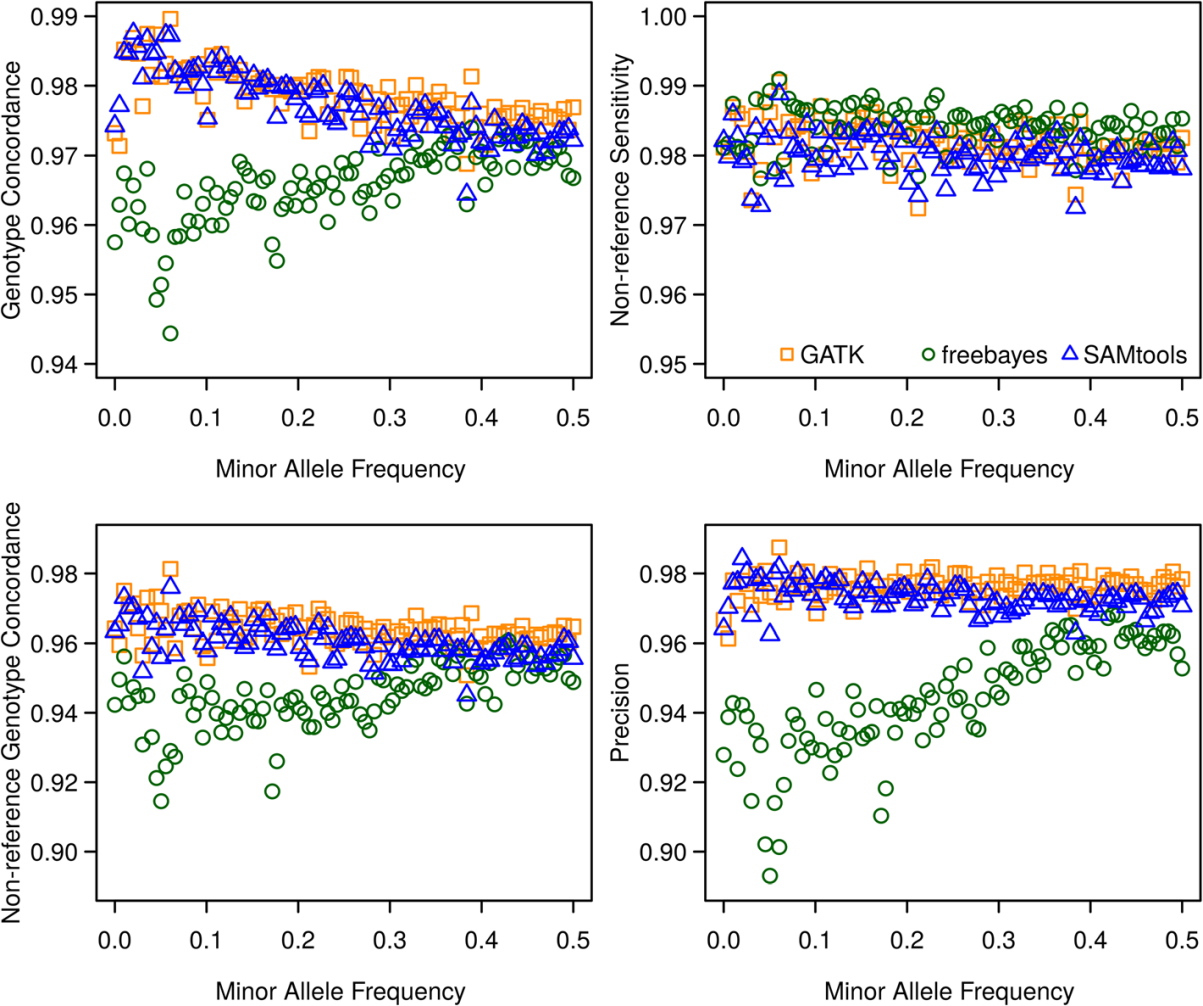
Comparison of the genotype concordance, non-reference sensitivity, non-reference genotype concordance and precision of GATK, freebayes and SAMtools over various minor allele frequency bins. SNPs were binned into 100 groups according to their array-derived MAF. The mean of each metric was calculated within each minor allele frequency bin. The statistics of different genotype concordance metrics were measured according to Linderman et.al [20]. The orange squares represent variant caller GATK. The green circles stand for variant caller freebayes. The blue triangles stand for variant caller SAMtools

Except the number of SNPs shared by different variant callers, we also compared GC, NRS, NRC, and precision of different callers as suggested in DePristo et al. and Linderman et al. [20, 38]. Different quality measures are shown in Table 1 and Additional file 6. In general terms, we obtained very high values (>0.9) for all metrics and all different callers, particularly for GATK and SAMtools. For 90 % of variants called by GATK all four metrics were simultaneously larger than 0.9, while this was the case for 88 % (75 %) of variants called with SAMtools (freebayes), which was mainly due to lower values in NRC and precision. The four different metrics which were binned into 100 groups according to their array-derived MAF plotted against array-derived MAF are shown in Fig. 2. In general, the similarity of metrics based on the SNPs called by GATK and SAMtools in different MAF bins was extremely high when compared to metrics based on SNPs called by freebayes. Results obtained with GATK and SAMtools were rather insensitive to MAF with the exception of GC, which showed a slight increase when MAF (<0.05) was low. Metrics for freebayes were in general lower (with the exception of NRS) and showed a slight increase with increasing MAF. The different properties of results of freebayes compared to GATK and SAMtools is likely due to (dis)similarities of the algorithms underlying the three programs. Although the priors are different, GATK [16] and SAMtools [15] use rather similar Bayesian methods for estimation of the posterior probability of the genotype and detection of variants relying on alignment. freebayes [30] also uses Bayesian methods to detect variants, but is haplotyped-based, in the sense that it calls variants based on the literal sequences of reads aligned to a particular target, not their precise alignment (https://github.com/ekg/freebayes). Although GATK and SAMtools have equivalent performances and both perform better than freebayes, the metrics used here relied on the accuracy of array-derived genotypes which were assumed to be the ‘true’ genotypes. Eventually existing genotyping errors may thus bias the results. Besides, SNPs on the array were selected to be almost evenly distributed across the genome and were preselected to match a certain MAF spectrum which differs from the MAF distribution present in the sequence [44], which also could bias the relative performance of variant callers if they differ in sensitivity to such patterns. Furthermore, the coverage of sequencing as a potential influence factor was not under consideration here. Linderman et al. [20] discovered that insufficient coverage could bias the GC metrics, particularly the NRS. Thus, freebayes might have more similar results to GATK or SAMtools if individuals were sequenced with a higher coverage.

**Table 1:** Genotype concordance metrics

	Genotype concordance						Non-reference sensitivity					
	GATK		Freebayes		SAMtools		GATK		Freebayes		SAMtools	
	No.SNPs	Mean ± SD	No.SNPs	Mean ± SD	No.SNPs	Mean ± SD	No.SNPs	Mean ± SD	No.SNPs	Mean ± SD	No.SNPs	Mean ± SD
≤0.1	23	0.03 ± 0.03	9	0.03 ± 0.03	3	0.08 ± 0.00	3	0.01 ± 0.02	9	0.04 ± 0.04	5	0.04 ± 0.04
≤0.2	18	0.14 ± 0.03	6	0.15 ± 0.03	6	0.15 ± 0.02	13	0.15 ± 0.03	10	0.14 ± 0.03	15	0.14 ± 0.03
≤0.3	12	0.25 ± 0.02	9	0.27 ± 0.02	9	0.25 ± 0.03	25	0.25 ± 0.03	7	0.24 ± 0.03	20	0.24 ± 0.03
≤0.4	13	0.33 ± 0.02	5	0.33 ± 0.01	18	0.35 ± 0.03	20	0.34 ± 0.02	11	0.34 ± 0.03	19	0.34 ± 0.03
≤0.5	22	0.45 ± 0.03	18	0.45 ± 0.03	24	0.45 ± 0.03	20	0.45 ± 0.02	14	0.45 ± 0.03	24	0.44 ± 0.03
≤0.6	42	0.55 ± 0.03	39	0.55 ± 0.04	43	0.55 ± 0.03	59	0.52 ± 0.03	89	0.51 ± 0.02	82	0.51 ± 0.03
≤0.7	73	0.65 ± 0.03	64	0.66 ± 0.03	62	0.66 ± 0.03	96	0.66 ± 0.02	105	0.66 ± 0.02	158	0.66 ± 0.03
≤0.8	110	0.76 ± 0.03	183	0.76 ± 0.03	123	0.76 ± 0.03	152	0.75 ± 0.02	150	0.75 ± 0.02	215	0.75 ± 0.02
≤0.9	679	0.87 ± 0.03	3828	0.88 ± 0.02	738	0.87 ± 0.03	1206	0.86 ± 0.03	1171	0.86 ± 0.03	1561	0.86 ± 0.03
≤1	50441	0.98 ± 0.02	46725	0.97 ± 0.02	50334	0.98 ± 0.02	49732	0.99 ± 0.02	49206	0.99 ± 0.02	49148	0.99 ± 0.02
	Non-reference genotype concordance						Precision					
≤0.1	55	0.02 ± 0.03	34	0.02 ± 0.03	35	0.02 ± 0.03	46	0.02 ± 0.04	28	0.01 ± 0.03	28	0.01 ± 0.03
≤0.2	18	0.13 ± 0.03	10	0.14 ± 0.03	23	0.14 ± 0.03	11	0.14 ± 0.03	12	0.15 ± 0.03	11	0.16 ± 0.03
≤0.3	24	0.26 ± 0.03	20	0.25 ± 0.03	24	0.25 ± 0.03	22	0.25 ± 0.03	22	0.25 ± 0.03	17	0.25 ± 0.03
≤0.4	32	0.34 ± 0.02	35	0.34 ± 0.02	43	0.35 ± 0.02	26	0.35 ± 0.03	21	0.34 ± 0.02	20	0.35 ± 0.03
≤0.5	32	0.43 ± 0.03	41	0.43 ± 0.03	38	0.43 ± 0.03	15	0.42 ± 0.02	36	0.43 ± 0.03	21	0.44 ± 0.03
≤0.6	144	0.52 ± 0.03	249	0.52 ± 0.03	168	0.52 ± 0.03	99	0.52 ± 0.03	161	0.52 ± 0.03	99	0.53 ± 0.03
≤0.7	245	0.66 ± 0.03	540	0.66 ± 0.03	297	0.65 ± 0.03	157	0.66 ± 0.03	499	0.66 ± 0.03	156	0.66 ± 0.03
≤0.8	445	0.75 ± 0.02	1725	0.76 ± 0.03	584	0.76 ± 0.02	270	0.75 ± 0.02	2246	0.76 ± 0.02	240	0.75 ± 0.02
≤0.9	3019	0.86 ± 0.03	6607	0.86 ± 0.03	3720	0.86 ± 0.03	1644	0.86 ± 0.03	6808	0.85 ± 0.03	1964	0.86 ± 0.03
≤1	47312	0.98 ± 0.03	41511	0.97 ± 0.03	46315	0.98 ± 0.03	49038	0.99 ± 0.02	40941	0.97 ± 0.03	48692	0.98 ± 0.03

The calculation based on array genotypes and corresponding sequence-based genotypes obtained with different variant callers at positions where SNPs from the array were available on chromosomes 3, 6 and 28

In this study, the analyses were mainly focused on the comparison of specific versions (namely the newest at the time point of performing the analyses) of different variant callers. Different versions of the same variant callers may result in a different set of variants for the same underlying sequencing data. We thus compared two different versions of freebayes. The older version of freebayes (version v9.9.2-22-gc283d6d) called 524,938 SNPs (29 %) more than the newer version (version v0.9.15-1-g076a2a2) based on the same data material on chromosomes 3, 6, and 28. Thus, our ranking of callers is only valid for the specific versions used here.

It needs to be mentioned that the only alignment tool used in this study was BWA with different variant callers to make sure that variants are called based on the same basic data sets, to ensure a fair comparison of the callers. However, each step listed in the pipeline (Additional file 7) affects the quality of the final SNP calls, including the alignment step. Besides, it is possible that different callers combined with different alignment tools may have a different performance, an aspect which was not investigated in this study. However, BWA is one of the widely used alignment tools, which is known to have a good performance as well [34]. Based on the results on the quality measures and regarding computation time (GATK which supports multiple threads was faster than SAMtools and freebayes, as shown in Additional file 8) and usefulness (i.e. quality, completeness and availability) of the software documentation, we decided to use the variants called by GATK as basis for the imputation part of this study. It turned out that there were 1,652,105 SNPs remaining on chromosomes 3, 6 and 28 totally in the brown layer chicken dataset for the imputation study. As mentioned before, there were different pipelines to deal with the whole-genome sequencing data with or without strictly filtering on genotype quality of each SNP and each individual. In this study, we did not filter genotype quality while enhancing the original genotype quality with Beagle 3.3.2 as was done in the study of Jansen et al [37] in order to use more SNPs in the analysis.

### Imputation accuracy

#### Leave-one-out cross-validation

**Fig. 3:**
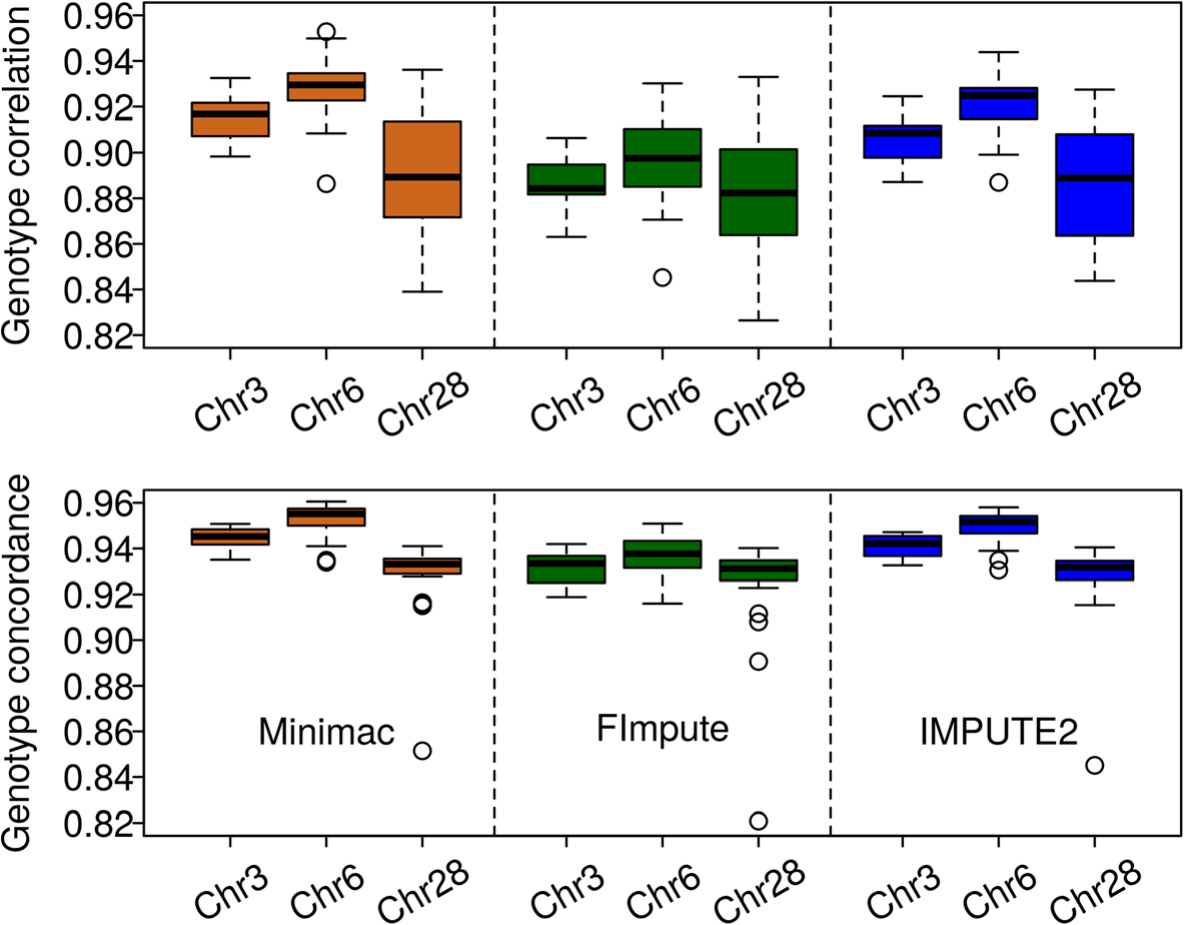
Imputation accuracy assessed by leave-one-out cross-validation. Genotype correlation (top panel) and genotype concordance (bottom panel) between the sequenced and imputed genotypes for 24 sequenced individuals with different imputing programs

A leave-one-out cross-validation was performed for each individual that was both sequenced and genotyped (i.e. 24 out of 25 sequenced individuals) to assess imputation accuracy from SNP array data to whole-genome sequence. For chromosomes 3, 6 and 28, the genotype correlation and concordance achieved by three different imputation packages (Minimac, FImpute and IMPUTE2) are shown in Fig. 3. Generally speaking, imputation accuracy assessed as correlation and concordance between imputed and sequence-derived genotypes within sequenced individuals was high with all imputation packages, with the performance of FImpute being slightly worse than the one of Minimac and IMPUTE2.

Over all three chromosomes, the average genotype correlation ± standard deviation between imputed and sequence-derived genotypes was 0.91 ± 0.028 for Minimac, 0.89 ± 0.028 for FImpute and 0.90 ± 0.027 for IMPUTE2. These results implied that also pedigree-free imputation software (Minimac and IMPUTE2) yielded accurate genotypes for whole sequence variants in this data set. Most of the sequenced individuals in this study were contemporaries in a commercial breeding program which controls for the level of relationship and inbreeding, thus pedigree relationship among these individuals was relatively low. This may explain why imputation programs based on pedigree algorithms, such as FImpute, have no advantage in this leave-one-out cross-validation strategy.

Over all three imputation programs, the average genotype correlations for SNPs on chromosomes 3 and 6 were quite similar. However, for chromosome 28, which is much smaller than the other two chromosomes studied, the average genotype correlation was slightly lower and the standard deviation was larger compared to chromosomes 3 and 6. In the study of Hancock et al. [45], it was also found that imputation accuracy tended to be better on larger chromosomes (i.e. chromosome 1) than on smaller chromosomes (i.e. chromosome 22) in the human genome, even when there was no significant difference between these two chromosomes in typical characteristics (e.g. SNP density). The results of genotype concordance had a similar tendency as genotype correlation, but with a smaller standard deviation, particularly for chromosome 28. Overall, the imputation accuracies of different programs were largely similar in this scheme, although FImpute again performed slightly worse than the other two on chromosomes 3 and 6.

Leave-one-out cross-validation is the only strategy of assessment of imputation quality that allows taking all SNPs from sequenced data into account when calculating measures like genotype correlation. However, it should be mentioned that in most cases in practice individuals to be imputed are descendants of the sequenced individuals, which was not the case in this leave-one-out setup. Assessing whether the sequence of offspring is correctly imputed can only be done if a sample of such offspring is actually sequenced, and such data are presently not available in sufficient quantities. Thus these results of this analysis should only be extrapolated with caution to the practically most relevant case of imputing sequence of current selection candidates based on sequenced founder animals.

#### Genotype conflicts in father-progeny pairs

**Fig. 4:**
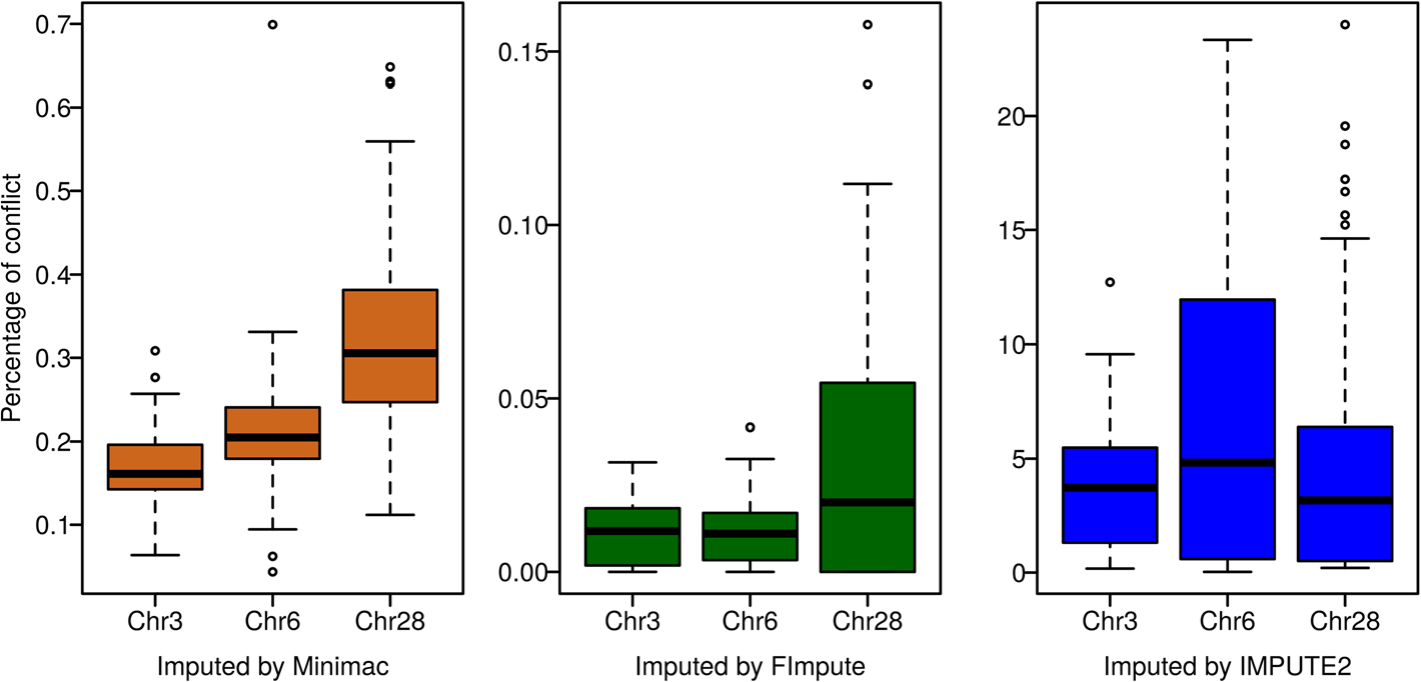
The percentage of genotype conflicts in father-progeny pairs. The conflicts were calculated within 952,826 (365,802, 37,556) imputed SNPs on chromosome 3 (6, 28) for 134 pairs of sequenced fathers and imputed progeny. Imputation was performed using Minimac (left), FImpute (middle) or IMPUTE2 (right)

Based on the available pedigree, it is possible to apply Mendelian rules to estimate the percentage of genotype conflicts for all SNPs in father-progeny pairs (i.e. progeny’s genotype is alternatively homozygous to father’s homozygous genotype) for an imputed progeny compared to each sequenced individual. There were 134 father-progeny pairs in the available pedigree for which the father was sequenced and the progeny was imputed. The number of progenies per father varied from 1 to 44. Comparisons of the imputation performance based on the father-progeny pair conflict (Fig. 4) show that FImpute (on average 0.01 %) outperformed Minimac and IMPUTE2 clearly, which should be expected, since pedigree information is used in FImpute while both other programs are pedigree-free algorithms. Furthermore, Minimac was still much better (on average 0.11 %) than IMPUTE2 which produced conflicts with 2.5 % of the imputed SNPs on average. When focusing on the performance of Minimac and FImpute on each chromosome, Minimac showed better performance on the larger chromosome 3 than on smaller chromosomes, likely due to the fact that there is more recombination on the micro-chromosomes which can result in less LD [46]. The results of FImpute for the three chromosomes were similar, in spite of the fact that the percentage of conflicts was slightly higher on chromosome 28 than on the other two.

#### Imputation accuracy for randomly masked 1000 SNPs in genotyped individuals

**Fig. 5:**
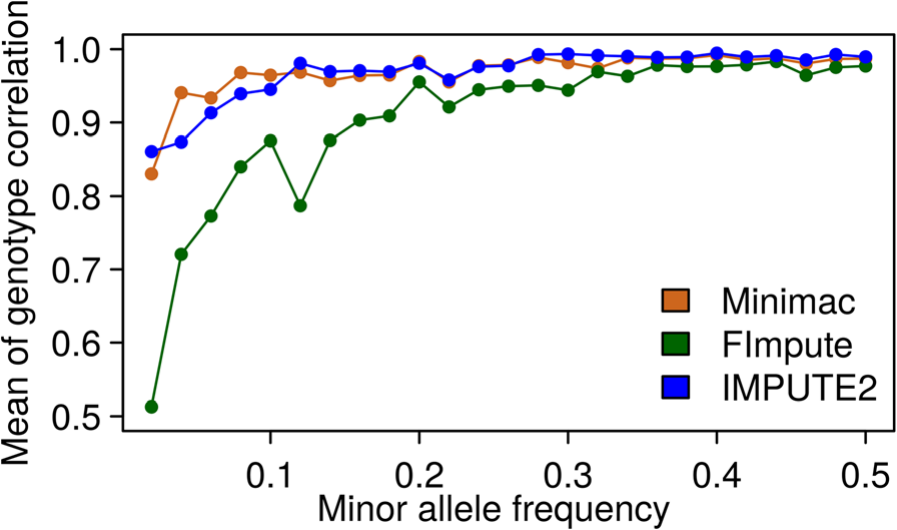
Mean of imputation accuracy of different software against minor allele frequency among 5 replications. SNP were binned by their sequence-derived MAF

In this scenario, the quality of imputation was assessed by the correlation between imputed and masked true genotypes per individual and/or per SNP. The average imputation accuracy of different software programs plotted for MAF bins is shown in Fig. 5. Correlation between imputed and true genotypes per SNP over all individuals was calculated. For this, all SNPs randomly masked in all 5 replicates were binned based by their sequence-derived MAF, and the average correlation in each bin was assessed. In general, the imputation accuracy (± S.D.) of FImpute was lower (0.90 ± 0.11) compared to Minimac (0.97 ± 0.033) and IMPUTE2 (0.97 ± 0.036). FImpute performed particularly poor for SNPs with a MAF smaller than 0.2. Imputation accuracies from Minimac and IMPUTE2 were comparatively stable with different MAFs, with a small reduction when MAF was low (<0.1). Our results are in general agreement with several previous studies (e.g. in cattle [9, 47, 48] or human [49, 50]) which also found that imputation accuracy decreased rapidly when MAF is low with different imputation software packages. Hence, the ability to accurately impute SNPs with low MAF is one of the most important criteria to assess the imputation programs. Usage of a diverse reference population may increase the imputation accuracy of rare variants [50–52], however, the computational burden also increases with the increase of the size of the reference population [31, 49]. Thus, the trade-off between the imputation accuracy and imputation efficiency needs to be considered.

**Fig. 6:**
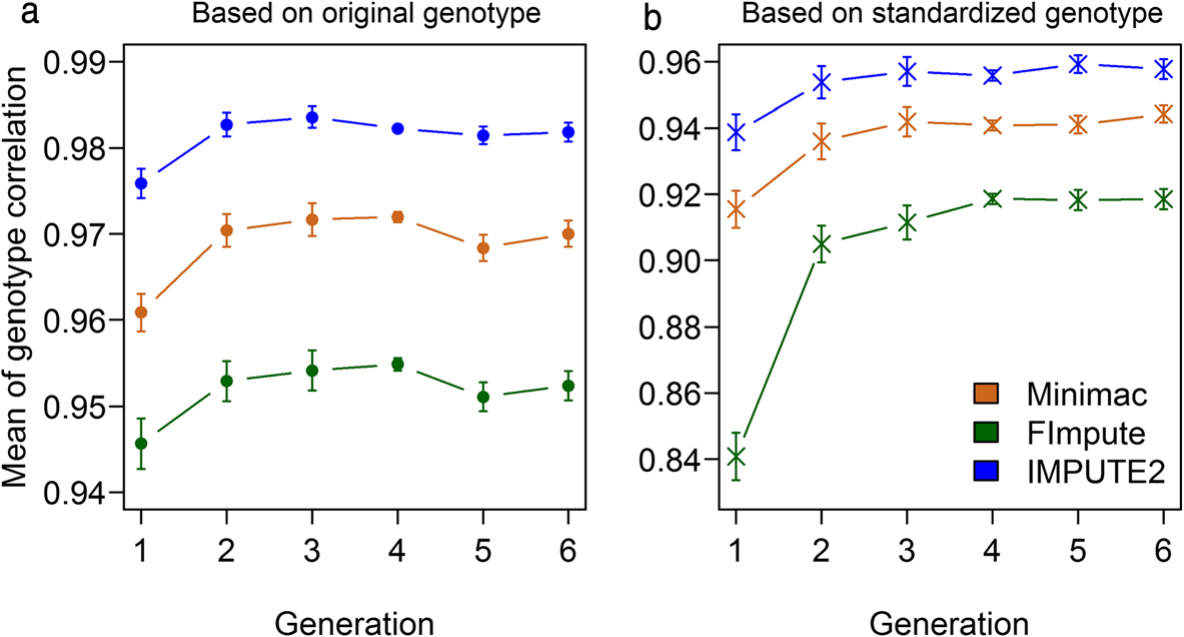
Imputation accuracy with 95 % CI of masked SNPs in different generations obtained with different imputation software package. The imputation accuracy is the correlation between the sequenced and imputed genotypes which were masked as dummy genotypes on 3 chromosomes (3, 6 and 28) with 5 replications. Imputation accuracy measured as the correlation between original imputed and original true genotype per individual is shown in (**a**), while imputation accuracy measured as the correlation between standardized imputed and standardized true genotype per individual is shown in (**b**)

As imputation accuracy depends (amongst others) on the relationship between reference individuals and individuals to be imputed, the trend of imputation accuracy when there was a different number of generations between reference and validation individuals was investigated. The relationship between sequenced individuals and genotyped individuals, which was estimated as the percentage of genotyped individuals having a high relationship ≥ 0.25 (or 0.5) with at least one of sequenced individual is shown in Additional file 9. Imputation accuracy with 95 % confidence interval obtained for individuals from different generations with different imputation programs is shown in Fig. 6. Imputation accuracy measured as the correlation between original imputed and original true genotype per individual is shown in Fig. 6a, while imputation accuracy measured as the correlation between standardized imputed and standardized true genotype per individual is shown in Fig. 6b. Generally, imputation accuracies for all three programs based on standardized genotype were lower than based on original genotype with larger standard deviation; however, the tendency of imputation accuracy along generations was the same for both measures. In the scenario with original genotype, comparing the three imputation software studied here, IMPUTE2 showed the highest genotype correlation for individuals from all generations, while FImpute showed the lowest genotype correlation. From generations 1 to 3, the average genotype correlation increased slightly, while from generations 4 to 6 hardly any trend was observed. However, there was no significant difference between adjacent generations while there was significant increase when comparing generation 1 to generations 4, 5 and 6 respectively for Minimac and IMPUTE2, and there was a significant increase between generation 1 and 4 for FImpute. In the scenario with standardized genotype, there was a significant increase between generation 1 and generation 2. These results suggested that imputing SNP array data up to sequence level is possible with high accuracy even across several generations. Our results thus confirm results from a previous study [25] which suggested that imputation quality did not deteriorate when the imputed population was three generations away from the sequencing population. It should be mentioned that the data we used here were from a closed line (Qanbari et al. [53] estimated the effect population size (*N*_*e*_) for individuals from a commercial brown layer line cross and found a recent *N*_*e*_ of 70) and the results may differ in more open populations with higher effective population size, migration or variability in mating schemes.

## Conclusions

Based on data from 50 sequenced individuals from two layer lines, we compared the performance of three variant callers for a subset of SNPs (~50 k) that were available from whole-genome sequencing and SNP array in 24 out of 1081 individuals that were both fully sequenced and genotyped with the 580 k array. Results showed that a high proportion of SNP calls had high values in different measures of quality (amongst others genotype concordance and non-reference sensitivity) with all variant callers. GATK showed a slightly better performance than SAMtools and freebayes. We further demonstrated that three commonly used imputation programs were capable of imputing from SNP array data up to whole-genome level in a brown layer line based on a small number of sequenced individuals with substantial imputation accuracy, even across several generations. FImpute performed slightly worse than Minimac and IMPUTE2 in terms of genotype correlation, especially for SNPs with low minor allele frequency, while it yielded the lowest numbers of Mendelian conflicts in available father-progeny pairs. Imputation accuracy was lower for rare SNPs than for common SNPs, which confirmed previous results in other species. Overall, sequence imputation from a very limited number of sequenced individuals appears to yield reasonably accurate results in closed breeding populations as available in many nucleus breeding programs.

## Availability of supporting data

The reference genome used for alignment was taken from a public database and is available for download from UCSC genome browser (http://hgdownload.soe.ucsc.edu/downloads.html#chicken). PLINK binary files containing genotype and map information of all variants on chromosomes 3, 6 and 28 detected by GATK in the 50 sequenced individuals are available at doi: 10.6070/H47H1GKK.

## Additional files

Additional file 1:
**Number of individuals in each generation.** (DOCX 17 kb) Additional file 2:
**Pipeline for how to improve the genotype quality and phasing.** (PDF 102 kb) Additional file 3:
**Different genotype concordance metrics.** 0, 1, and 2 is the number of non-reference allele. NA is SNPs which did not pass the filtering or missing genotype. This graph was adjusted and modified based on definition of DePristo et al. [38] and Linderman et al. [20]. (PNG 69 kb) Additional file 4:
**Read and coverage.** (XLSX 17 kb) Additional file 5:
**Number of variants identified by GATK, SAMtools and freebayes in the layer chicken genome.** (XLSX 13 kb) Additional file 6:
**Boxplot of genotype concordance, non-reference sensitivity, non-reference genotype concordance and precision calculated based on array genotypes and corresponding sequence-based genotypes obtained with different variant callers (GATK, freebayes, SAMtools) at positions where SNPs from the array were available on chromosomes 3, 6 and 28 (~50 k).** The statistics of different genotype concordance metrics were measured according to Linderman et.al [20]. (PNG 239 kb) Additional file 7:
**Pipeline.** (PDF 106 kb) Additional file 8:
**Running time.** (XLSX 11 kb) Additional file 9:
**Percentage of genotyped individuals having a high relationship ≥ 0.25 (or 0.5) with at least one of sequenced individual.** (PNG 65 kb)

## Abbreviations

SNPs: Single nucleotide polymorphisms
MAF: Minor allele frequency
INDELs: Insertion and deletion
DP: Depth of coverage
MQ: Mapping quality
MQM: mean mapping quality of observed alternate alleles
MQMR: mean mapping quality of observed reference alleles
GC: Genotype concordance
NRS: non-reference sensitivity
NRC: Non-reference genotype concordance
LD: Linkage disequilibrium
CI: Confidence interval
SAM: Sequence Alignment/Map format
BAM file: binary version of SAM file
