# Supplementary material for: Comparison among three variant callers and assessment of the accuracy of imputation from SNP array data to whole-genome sequence level in chicken
Source: BMC Genomics. 2015 Oct 21;16:824. doi: 10.1186/s12864-015-2059-2 (PMC4618161; doi:10.1186/s12864-015-2059-2)
Supplement: Additional file 1: — Number of individuals in each generation. (DOCX 17 kb) [file 12864_2015_2059_MOESM1_ESM.docx]

Table: Number of individuals with array data or whole genome sequencing data in each generation

| Generation | 1 | 2 | 3 | 4 | 5 | 6 | Total |
| --- | --- | --- | --- | --- | --- | --- | --- |
| Array data | 86 | 61 | 66 | 642 | 114 | 112 | 1,081 |
| Sequence data | 22 | 1 | 2 | - | - | - | 25 |

Table: Number of individuals in pedigree across each generation

| Generation | #individuals |
| --- | --- |
| -6 | 3 |
| -5 | 61 |
| -4 | 86 |
| -3 | 99 |
| -2 | 119 |
| -1 | 151 |
| 1 | 218 |
| 2 | 224 |
| 3 | 293 |
| 4 | 692 |
| 5 | 202 |
| 6 | 112 |
