# Supplementary figures and images for: Comparison among three variant callers and assessment of the accuracy of imputation from SNP array data to whole-genome sequence level in chicken
Source: BMC Genomics. 2015 Oct 21;16:824. doi: 10.1186/s12864-015-2059-2 (PMC4618161; doi:10.1186/s12864-015-2059-2)

# Pipeline of strategy for genotype quality enhancement

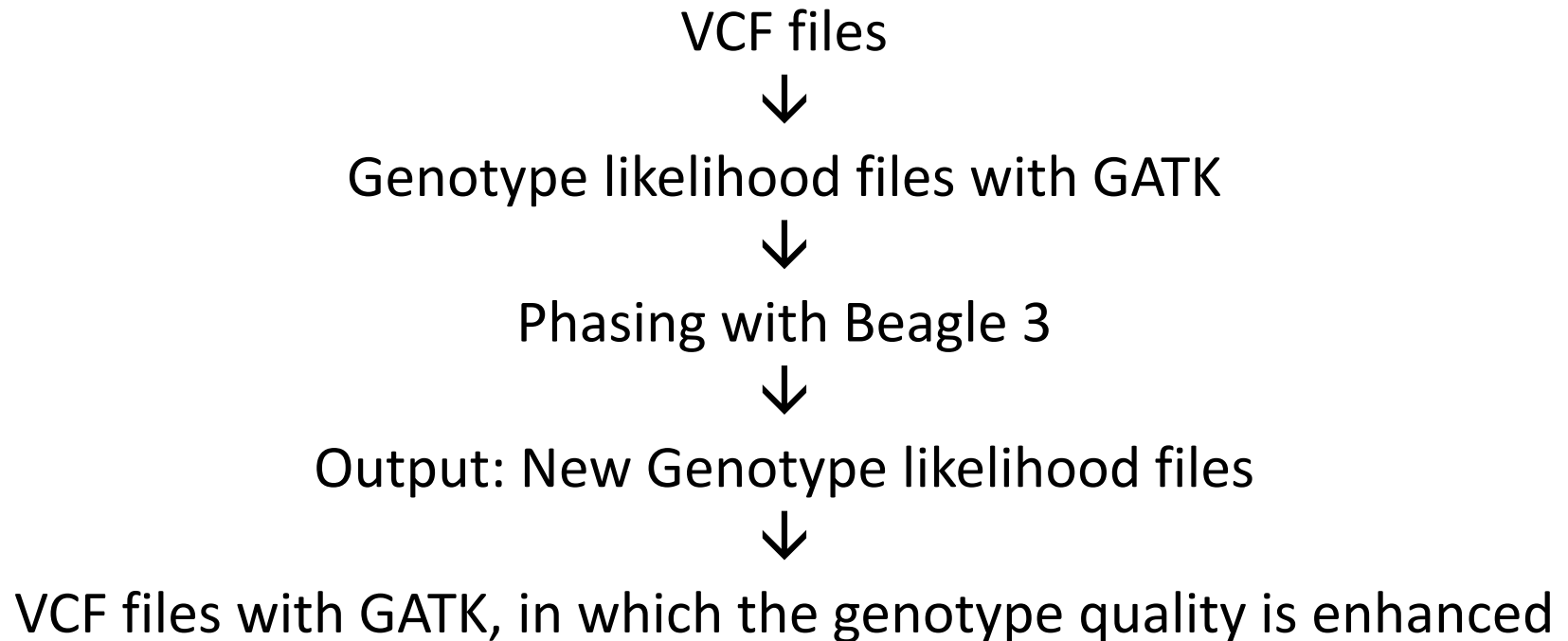

Supplement: Additional file 2: — Pipeline for how to improve the genotype quality and phasing. (PDF 102 kb) [file 12864_2015_2059_MOESM2_ESM.pdf]

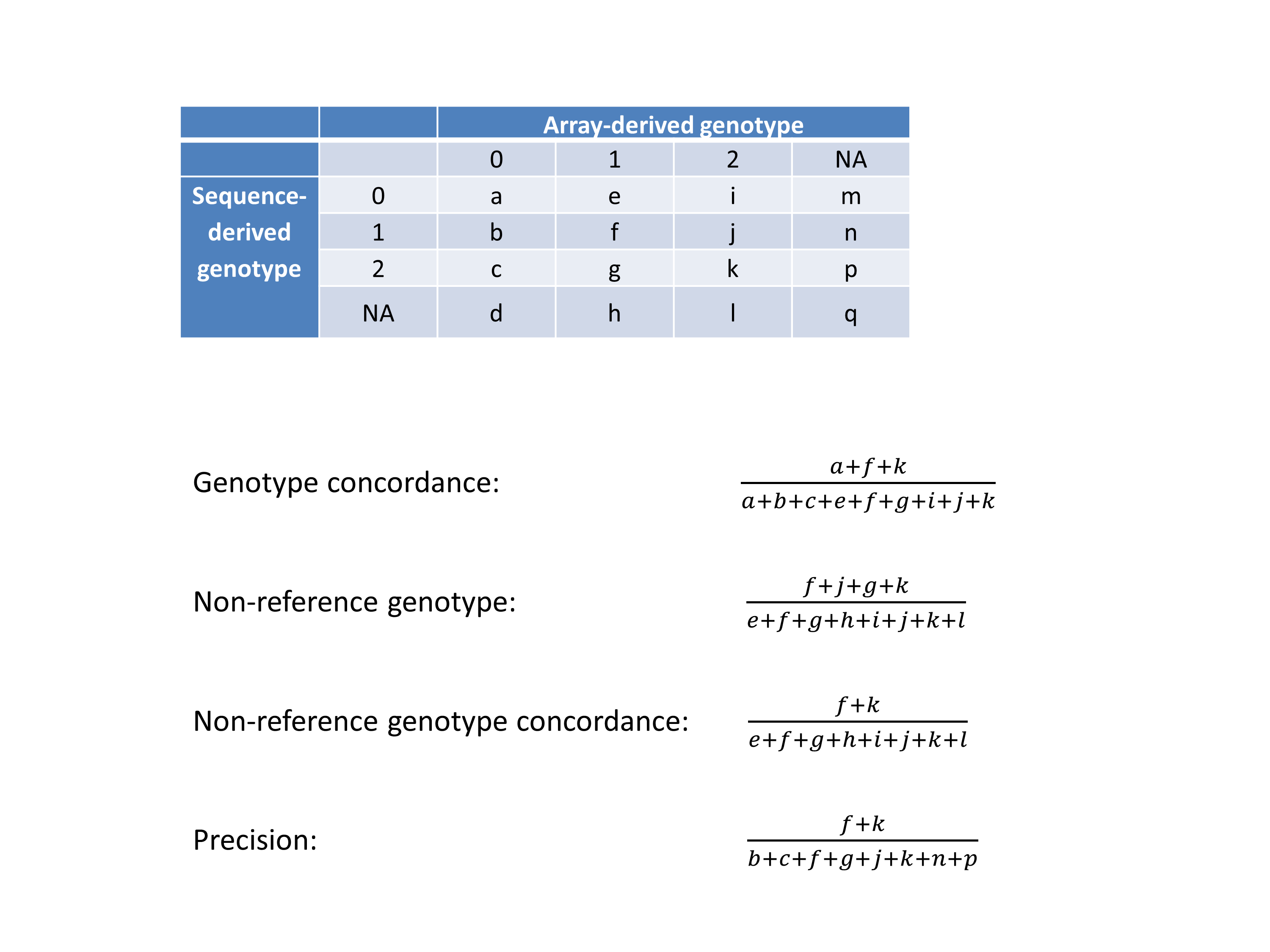

Supplement: Additional file 3: — Different genotype concordance metrics. 0, 1, and 2 is the number of non-reference allele. NA is SNPs which did not pass the filtering or missing genotype. This graph was adjusted and modified based on definition of DePristo et al. [38] and Linderman et al. [20]. (PNG 69 kb) [file 12864_2015_2059_MOESM3_ESM.png]

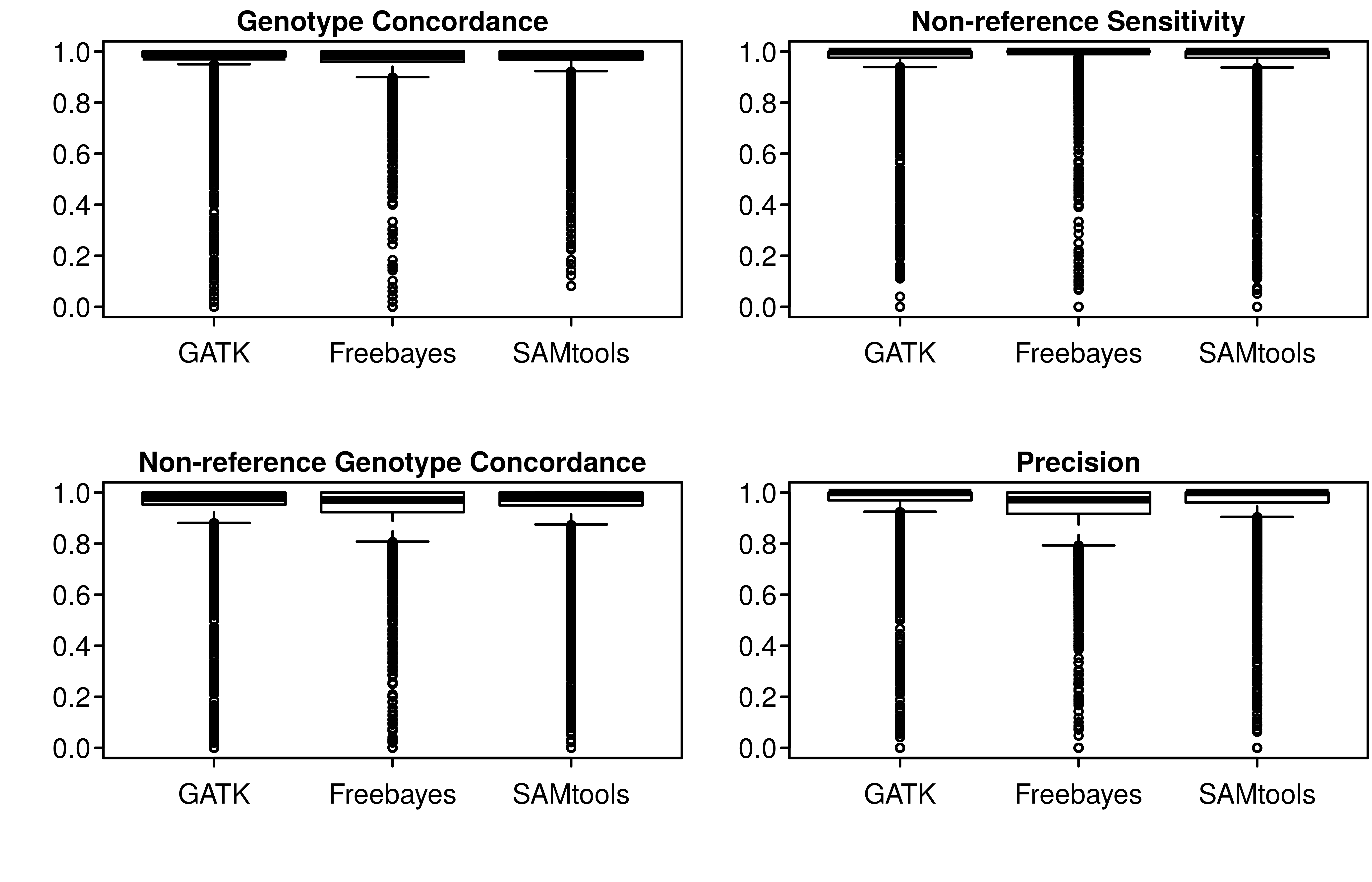

Supplement: Additional file 6: — Boxplot of genotype concordance, non-reference sensitivity, non-reference genotype concordance and precision calculated based on array genotypes and corresponding sequence-based genotypes obtained with different variant callers (GATK, freebayes, SAMtools) at positions where SNPs from the array were available on chromosomes 3, 6 and 28 (~50 k). The statistics of different genotype concordance metrics were measured according to Linderman et.al [20]. (PNG 239 kb) [file 12864_2015_2059_MOESM6_ESM.png]

## Pipeline

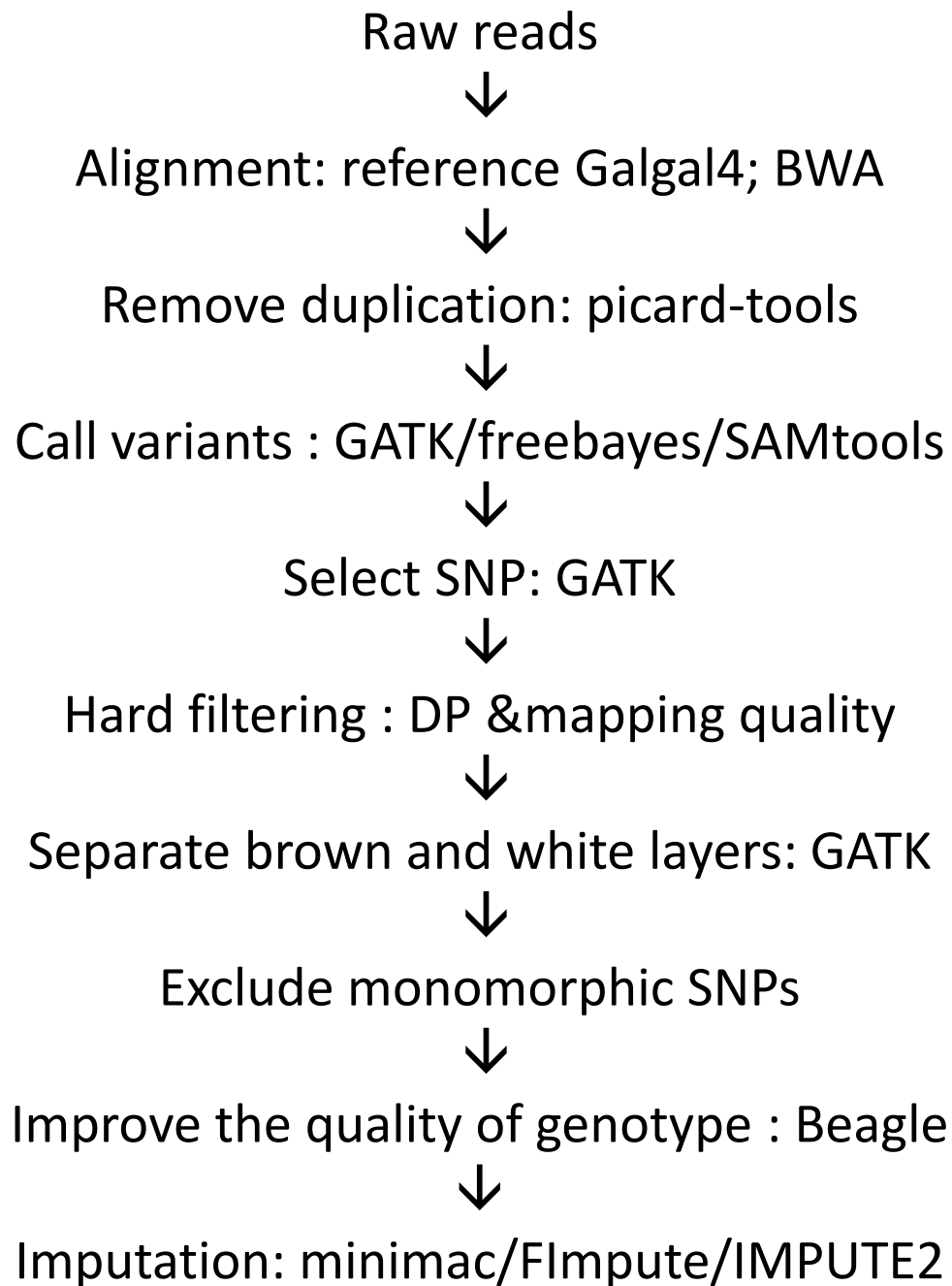

Supplement: Additional file 7: — Pipeline. (PDF 106 kb) [file 12864_2015_2059_MOESM7_ESM.pdf]

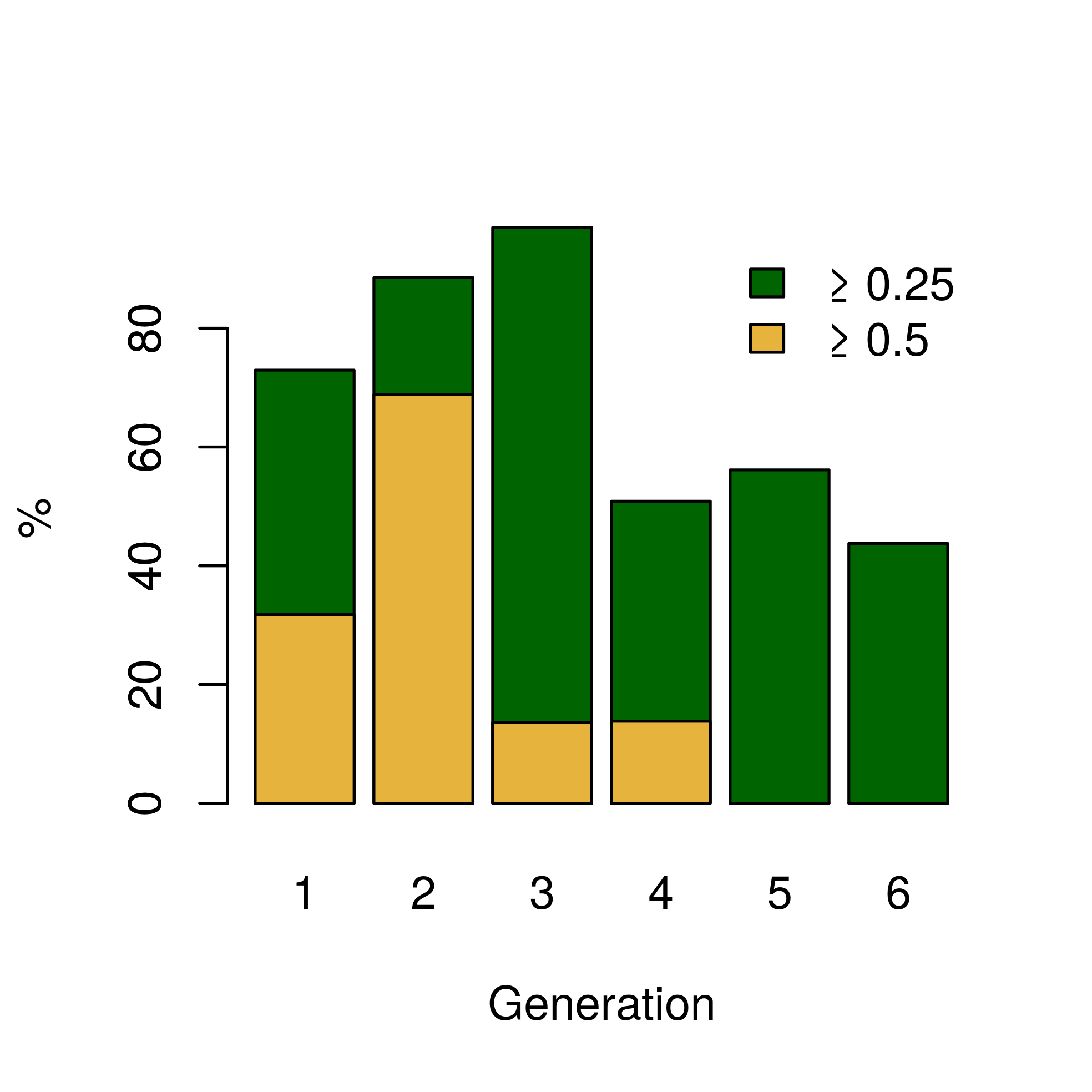

Supplement: Additional file 9: — Percentage of genotyped individuals having a high relationship ≥ 0.25 (or 0.5) with at least one of sequenced individual. (PNG 65 kb) [file 12864_2015_2059_MOESM9_ESM.png]
